# Intergenerational Contact Experiences and Their Relation to Ageism as a Multidimensional Construct

**DOI:** 10.1093/geroni/igab046.684

**Published:** 2021-12-17

**Authors:** Tim Kuball, Georg Jahn, Claas Pollmanns

**Affiliations:** Chemnitz Technical University, Chemnitz, Sachsen, Germany

## Abstract

Research on intergroup contact suggests that negative contact experiences affect cognitive representations such as stereotypes more strongly than positive contact experiences. To comprehensively examine the full effect of intergroup contact, the valence of the contact experience as well as the affective and cognitive dimensions of prejudice should be assessed. In ageism research, previous studies typically focused only on contact of positive valence and were limited to the perspectives of younger individuals on older adults. Primary objective of this study is to examine both positive and negative contact frequency and their relation to affective and cognitive dimensions of ageism from the perspectives of younger adults between the age of 18 and 25 (study 1) and older adults between the age of 60 and 92 (study 2). Consistent with previous research on intergroup contact, our results confirm that both types of contact were similarly predictive of affective facets of prejudice. However, only in study 2 that assessed older adults’ agreement with contemporary stereotypes about young men and women, negative compared to positive contact frequency proved to be a stronger predictor of the cognitive dimension of ageism. Our findings emphasize the importance of focusing on all dimensions of prejudice and highlight the need to consider the perspectives of young and old in ageism research.

